# A Case of Cerebral Amyloid Angiopathy With Recurrent Hemorrhagic and Ischemic Strokes Under Direct Oral Anticoagulant Therapy for Atrial Fibrillation

**DOI:** 10.7759/cureus.77679

**Published:** 2025-01-19

**Authors:** Junichi Uemura, Shinji Yamashita, Yoshiki Yagita, Takeshi Inoue

**Affiliations:** 1 Stroke Medicine, Kawasaki Medical School, General Medical Center, Okayama, JPN; 2 Stroke Medicine, Kawasaki Medical School, Kurashiki, JPN

**Keywords:** atrial fibrillation, caa-related inflammation, cerebral amyloid angiopathy, direct oral anticoagulant therapy, micro infarction

## Abstract

A 68-year-old male patient was admitted to our clinic with chronic atrial fibrillation (AF) and decreased consciousness. Diffusion-weighted magnetic resonance imaging of the brain revealed areas of high signal intensity, T2* cortical microbleeds (MBs), and cortical superficial siderosis. Cerebral amyloid angiopathy (CAA) with micro-infarction was diagnosed, and direct oral anticoagulant (DOAC) therapy was initiated. Due to increasing MBs and intracerebral hemorrhages, DOAC was discontinued. Subsequently, the micro-infarction and MBs abated, and the patient’s consciousness gradually improved. Catheter ablation was performed for AF, restoring sinus rhythm. DOAC can be harmful for CAA patients with AF linked to cortical hemorrhage. Thus, it is important to discontinue DOACs for micro-infarctions in cases of CAA with AF.

## Introduction

Cerebral amyloid angiopathy (CAA) is a disease characterized by repeated subcortical hemorrhages, microbleeds (MBs), and cognitive decline caused by the accumulation of amyloid beta (Aβ), primarily Aβ40, in the arterioles on the surface of the brain [1]. Patients with CAA often have atrial fibrillation (AF). For AF, direct oral anticoagulant (DOAC) is strongly recommended in patients with a CHAD2S score > 1 to prevent cardioembolic stroke. However, DOAC may increase the risk of cerebral hemorrhage in patients with CAA [2-4].

In the present case, the patient presented with CAA with AF. We continued DOAC because of increased microinfarctions. However, the symptoms worsened, and cerebral hemorrhage occurred. Thus, we discontinued DOAC, and his symptoms gradually improved without recurrence of microinfarctions or intracerebral hemorrhage. The clinical decision to use DOAC or not in patients with CAA and AF is multi-faceted. In this report, we discuss the pros and cons of using DOAC and suggest alternative treatments to prevent recurrent cerebral infarction in CAA patients with AF.

## Case presentation

A 68-year-old man who complained of a headache was admitted to a hospital. The patient had a history of hypertension and AF and had been taking apixaban 10 mg/day for the past year. The patient also had no history of smoking or drinking and no family history of stroke. A brain computed tomography (CT) scan revealed small intracerebral hemorrhage in the right frontal subcortex, while brain magnetic resonance imaging diffusion-weighted imaging (MRI-DWI) showed a small microinfarction in the left frontal hemisphere. The patient received a diagnosis of a subcortical hemorrhage and cardioembolic stroke, and was treated with the antihypertensive drug amlodipine 5 mg/day; apixaban was discontinued. He showed improvement by day 2, and apixaban at 10 mg/day was restarted on day 14. On day 21, the patient's level of consciousness decreased. It was determined that specialized treatment was necessary, and the patient was transferred to our hospital.

The patients vitals were as follows: blood pressure was 126/70 mmHg, pulse rate was AF 75 bpm, respiratory rate was 15 breaths per minute, body temperature was 36.6℃, and SpO_2_ was 97% at room air. Neurological examination revealed a consciousness level of JCS-I-2, no aphasia, apraxia, and short-term memory impairment. His cranial nerves, motor system, sensory system, coordinated movement, and deep reflexes were normal.

Laboratory examinations indicated that the patient exhibited a normal blood count. The patient’s erythrocyte sedimentation rate (ESR) was 17 mm/h, and coagulation and fibrinolysis tests showed no abnormalities in prothrombin time, activated partial thromboplastin time, fibrinogen, or antithrombin III. However, the patient’s D-dimer level was elevated at 1.2 µg/dL. Additionally, blood biochemistry test results revealed slightly elevated C-reactive protein (CRP) and B-type natriuretic peptide levels of 0.19 mg/dL and 87.6 pg/mL, respectively. Tests for anti-nuclear, anti-cardiolipin, anti-β2GP1, antineutrophil cytoplasmic antibodies, and lupus anticoagulants showed normal results (Table 1).

**Table 1:** Laboratory examinations at presentation WBC: white blood cell, RBC: red blood cell, ESR: Erythrocyte sedimentation rate, PT-INR: Prothrombin Time and International Normalized Ratio, APTT: Activated partial thromboplastin time, AST: Aspartate aminotransferase, ALT: Alanine aminotransferase, LDH: Lactate dehydrogenase, ALP: Alkaline phosphatase, γ-GTP: Gamma-glutamyl transferase, ChE: Cholinesterase: CPK: Creatine phosphokinase, Cr: Creatinine, BUN: Blood urea nitrogen test, PR3-ANCA: Proteinase 3-specific antineutrophil cytoplasmic antibody, MPO-ANCA: Myeloperoxidase-specific antineutrophil cytoplasmic antibody

Test name	Resuts	Normal Ranges
WBC	4600/µL	3300-8600/µL
RBC	4.81*10⁶/µL	4.35-5.55*10⁶/µL
Platelets	194*10³/µL	158-348*10³/µL
ESR	17mm/h	1-mm/h
PT-INR	0.99	0.85-1.13
APTT	29.4s	24-34s
Fibrinogen	278mg/dL	200-400mg/dL
D-dimer	1.2μg/dL	<1.0μg/dL
Total Protein	7.1g/dL	6.6-8.1g/dL
Albumin	4.1g/dL	4.1-5.1g/dL
Total Bilirubin	1.2mg/dL	0.4-1.5mg/dl
AST	23U/L	13-30U/L
ALT	27U/L	10-42U/L
LDH	183U/L	124-222U/L
ALP	103U/L	38-113U/L
γ-GTP	57U/L	124-222U/L
ChE	279U/L	240-486U/L
Amylase	47U/L	44-132U/L
CPK	68U/L	59-248U/L
Total Colesterol	192mg/dL	142-248mg/dL
Triglyceride	70mg/dL	40-149mg/dL
Cr	0.78mg/dL	0.65-1.07mg/dL
BUN	8mg/dL	8-20mg/dL
Sodium	140mmol/L	138-145mmol/L
Potassium	3.8mmol/L	3.6-4.8mmol/L
Chloride	102mmol/L	101-108mmol/L
C-reactive protein	0.19mg/dL	<0.14mg/dL
B-type natriuretic peptide	87.6pg/mL	0-18.4pg/mL
Anti-ss-DNA IgG antibodies	16AU/mL	0-25AU/mL
Anti-ds-DNA IgG antibodies	<10IU/mL	0-12IU/mL
Anticardiolipin IgG antibodies	10.1U/ml	0-12.3U/ml
Antiβ2GP1IgG antibodies	<=1.2U/ml	<3.5U/ml
Lupus anticoagulants (dRVVT)	1	0-1.2
PR3-ANCA	<1.0U/ml	<3.5U/ml
MPO-ANCA	<1.0U/ml	<3.5U/ml

Cerebrospinal fluid (CSF) analysis showed a slightly elevated monocytic cell count of 10/µL (100% mononuclear cells) and slightly elevated protein levels of 108 mg/dL, with a glucose level of 43 mg/dL and IgG index of 0.6. General culture, acid-fast bacillus culture, and cytology results were negative (Table 2).

**Table 2:** Cerebrospinal fluid analysis

Test Name	Results	Normal Ranges
Cell count	Monocyte 10/µg	0-5/µg
Protein	108mg/dl	10-40mg/dl
Glucose	43mg/dl	50-75mg/dl
IgG index	0.6	0.3-0.7
Oligoclonal band	Negative	Negative
Bacterial Culture	Negative	Negative
Tuberculosis culture	Negative	Negative
Cytology	Negative	Negative

**Figure 1:**
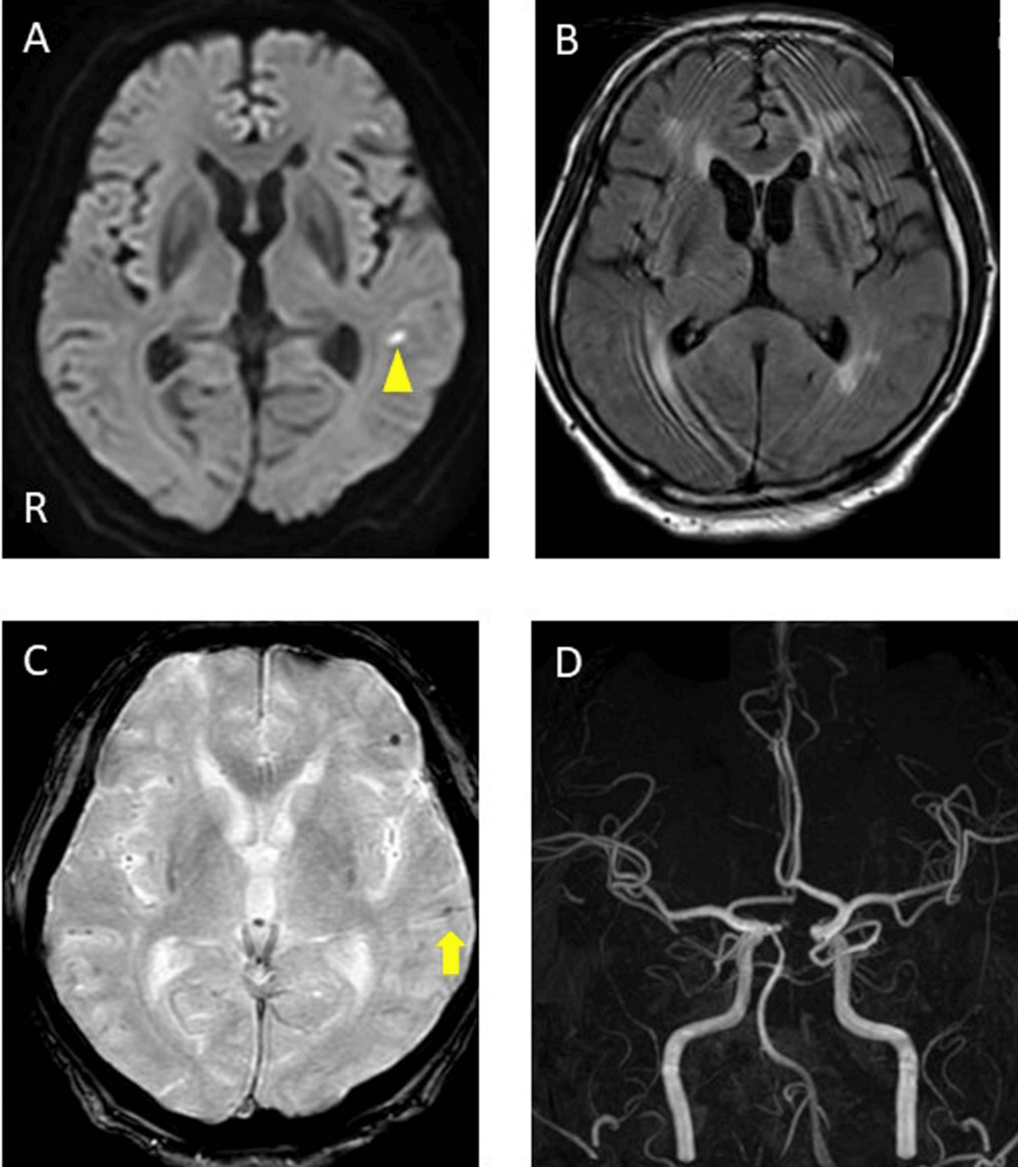
Brain magnetic resonance imaging (MRI) and magnetic resonance angiography (MRA) on day 21 A: Diffusion-weighted imaging (DWI) (3T, b =1000 s/mm², horizontal slice). A small high-signal area was observed in the left temporal lobe (yellow triangle). B: Fluid-attenuated inversion recovery image (FLAIR) showed white matter lesions on both sides. C: T2* imaging demonstrated new microbleeds in the left sulcus (yellow triangle) and cortical superficial siderosis in the left temporal lobe (yellow arrow). D: MRA showed no stenosis or occlusion of the main arteries. R: right

A 12-lead electrocardiogram (ECG) showed AF (76 bpm) and chest radiography indicated a cardiothoracic ratio of 52%, with clear lungs. Carotid ultrasound results were normal, transcranial ultrasound indicated a negative right-to-left shunt, and lower limb venous echography showed no obvious deep vein thrombosis. Transthoracic echocardiogram revealed bilateral atrial enlargement, ejection fraction of 52%, and no intracardiac thrombus. Brain MRI-DWI indicated a small high signal in the left frontal and temporal sub-cortical areas. Fluid-attenuated inversion recovery imaging demonstrated Fazekas Grade III periventricular hyperintensity, while T2* imaging demonstrated new MBs in the left sulcus and new cortical superficial siderosis (cSS) in the left temporal lobe. Brain magnetic resonance angiography showed no intra-arterial stenosis (Figure 1A-1D). Cerebral three-dimensional CT angiography showed no significant abnormal blood vessels or cerebral venous obstruction, and contrast CT from the trunk to the lower legs showed no evident malignant tumors, pulmonary embolisms, or deep vein thrombosis. Electroencephalography (EEG) showed no obvious epileptic form waves, and otherwise revealed slow waves.

**Figure 2:**
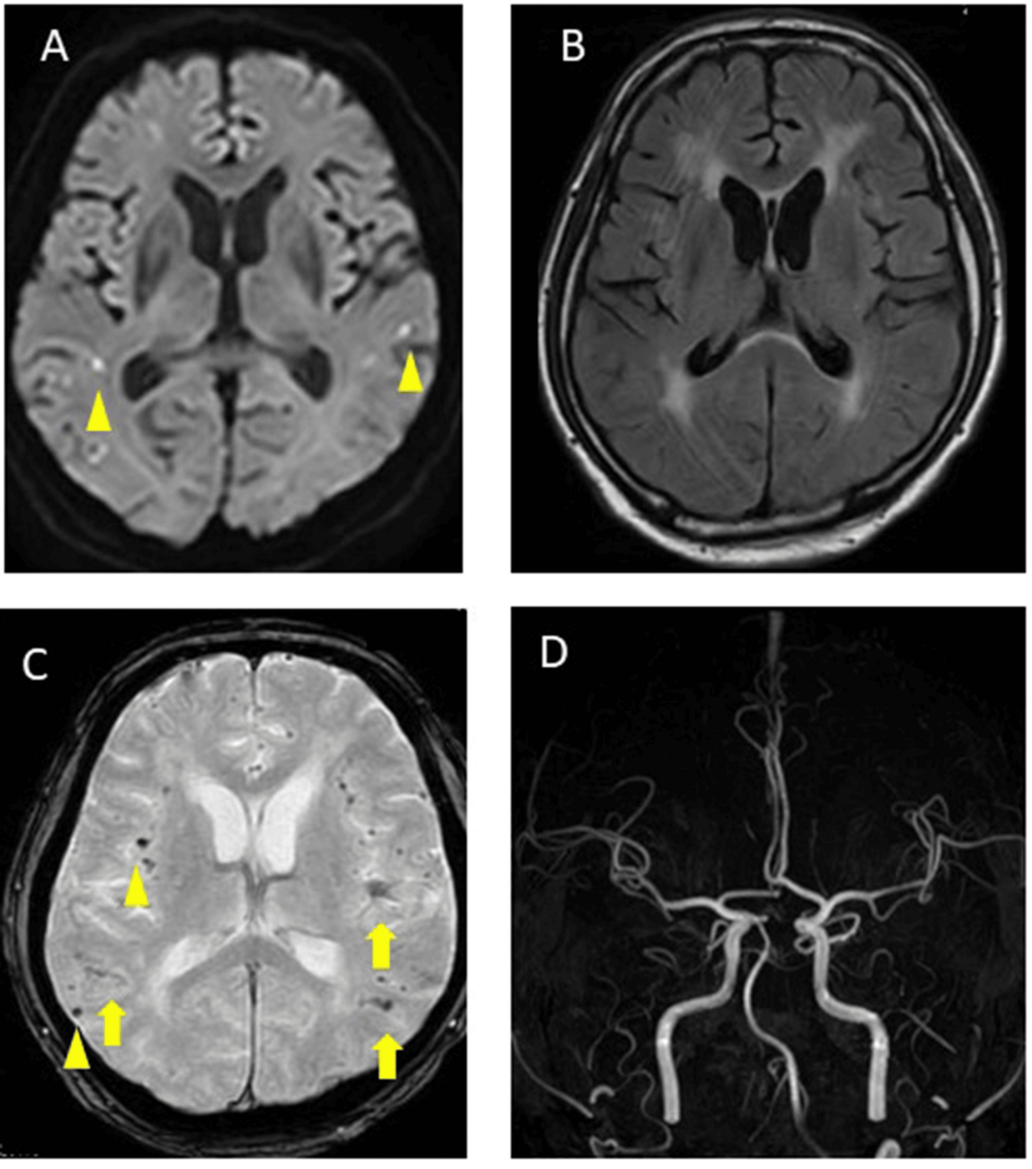
Brain magnetic resonance imaging and magnetic resonance angiography (MRA) on day 29 A: Diffusion-weighted imaging (DWI) showed hyperintensities in both parietal regions (yellow triangle). B: Fluid-attenuated inversion recovery image (FLAIR) showed no change in white matter lesions. C: T2* imaging demonstrated cortical superficial siderosis in the left frontal and both parietal sulci (yellow arrow). D: MRA showed no stenosis or occlusion in the major arteries.

The patient received a diagnosis of microinfarction with possible CAA according to the Boston Criteria 2nd Edition [1]. After admission, the treatment included edaravone 60 mg/day, amlodipine 10 mg/day, and olmesartan 40 mg/day, with apixaban 10 mg/day continued. However, the patient exhibited persistent consciousness disturbances, minimal spontaneous speech, and apathy. On day 29, the patient's level of consciousness suddenly decreased (JCS-III-100), accompanied by left conjugate deviation and right gaze disorder. A repeat brain MRI showed increasing DWI hyper intensities in both parietal regions, while T2* imaging demonstrated an increase in MBs in the right frontal parietal lobe and cSS in the left frontal and both parietal sulci (Figure 2A-2D). Arterial spin labeling showed no differences in blood flow between the left and right sides of the cerebral hemispheres. The patient’s level of consciousness improved immediately after the MRI, and EEG identified some slow waves; no obvious epileptiform activity was noted. On day 30, his Mini-Mental State Examination (MMSE) score was 3/30. As he suddenly lost consciousness, had conjugate deviation to the left, and had slow waves on EEG, systemic epilepsy with possible CAA was suspected and the patient was started on lacosamide at 100 mg/day. Subsequently, the patient's consciousness disorder, higher brain dysfunction, cognitive impairment, and speech disorder began to improve, and the MMSE score increased to 23/30 by day 61.

**Figure 3:**
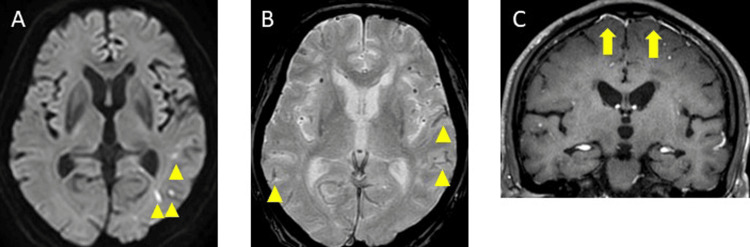
Brain contrast magnetic resonance imaging (MRI) and magnetic resonance angiography (MRA) on day 66 A: Diffusion-weighted imaging showed an increase in small high signals in the left parietal occipital lobe (yellow triangle). B: T2* imaging demonstrated an increase in cortical superficial siderosis in the right parietal, left frontal, and parietal sulcus (yellow triangle). C: A contrast-enhanced brain MRI on day 66 indicated contrast enhancement in the meninges (yellow arrow).

**Figure 4:**
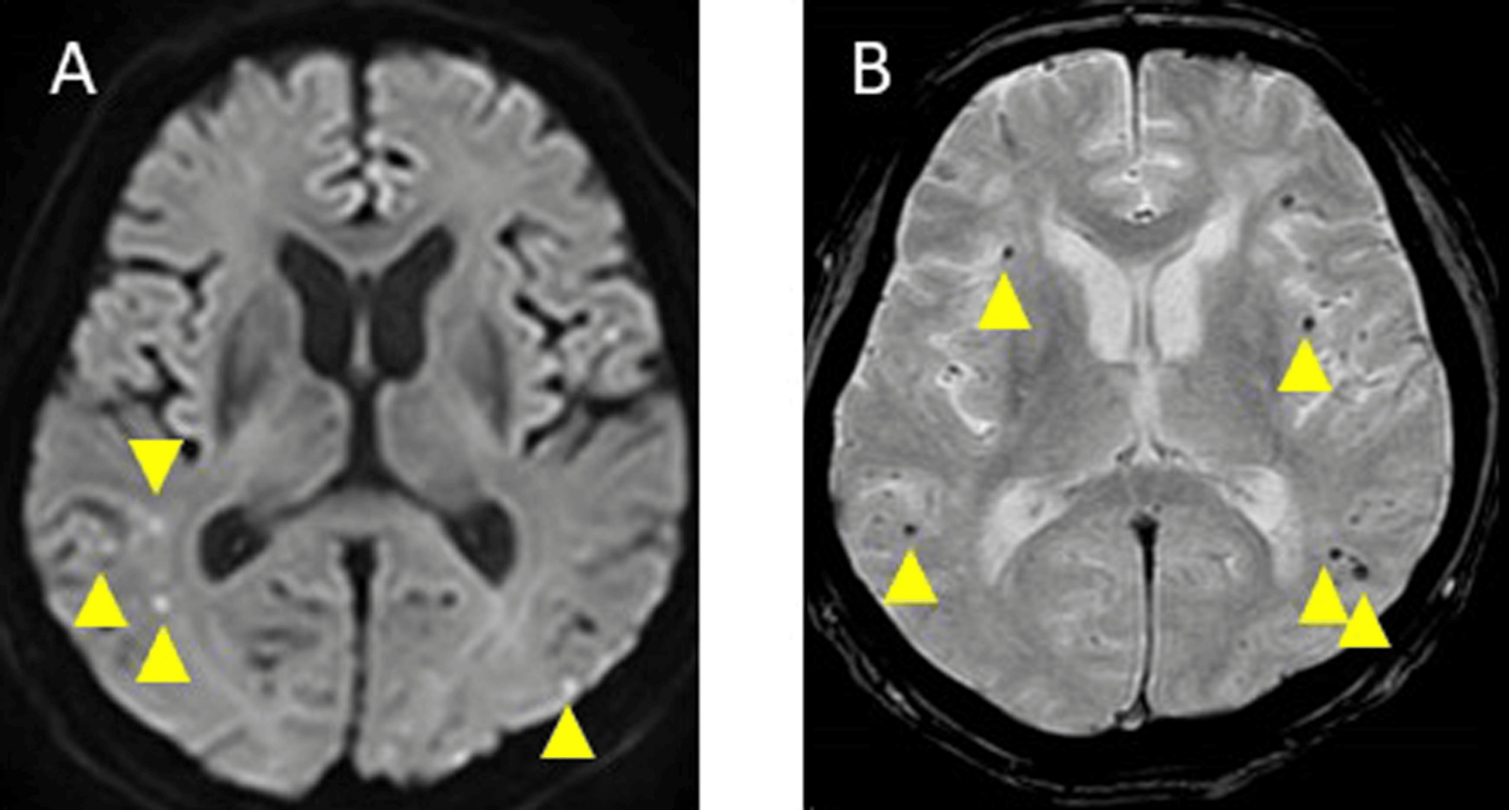
Brain magnetic resonance imaging diffusion-weighted imaging (DWI), and T2*, on day 73 A: MRI-DWI image showed an increase in small high signals in both occipital and parietal lobes (yellow triangle). B: T2* imaging demonstrated an increase in microbleeds in both frontal parietal lobes (yellow triangle).

**Figure 5:**
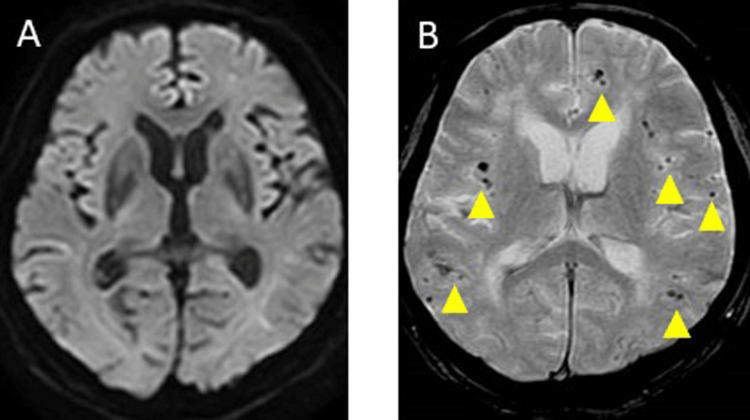
Brain magnetic resonance imaging diffusion-weighted imaging (DWI), and T2*, on day 88 A: MRI-DWI showed no increase of small high signals. B: T2* imaging demonstrated an increase in microbleeds in both frontal parietal lobes (yellow triangle).

Physical, occupational, and speech therapy were continued; however, on day 66, a follow-up MRI-DWI revealed an increase in the small high signals in the left parietal occipital lobe (Figure 3A), and T2* imaging showed an increase in the cSS in the right parietal, left frontal, and parietal sulcus (Figure 3B). A contrast-enhanced brain MRI indicated contrast enhancement in the meninges (Figure 3C). Rehabilitation was continued, and on day 73, a follow-up MRI-DWI revealed an increase in the small high signals in both the occipital and parietal lobes (Figure 4A), and T2* imaging demonstrated an increase in the MBs in both the frontal and parietal lobes (Figure 4B). He experienced gradual cognitive deterioration and was less receptive to rehabilitation. On day 80, a repeat MRI-DWI showed no increase of small high signals (Figure 5A), and T2* imaging revealed an increase in the MBs in both the frontal and parietal lobes (Figure 5B). He refused rehabilitation and as he was unable to continue hospitalization, he was discharged home with a modified Rankin Scale score of 3 at day 109.

**Figure 6:**
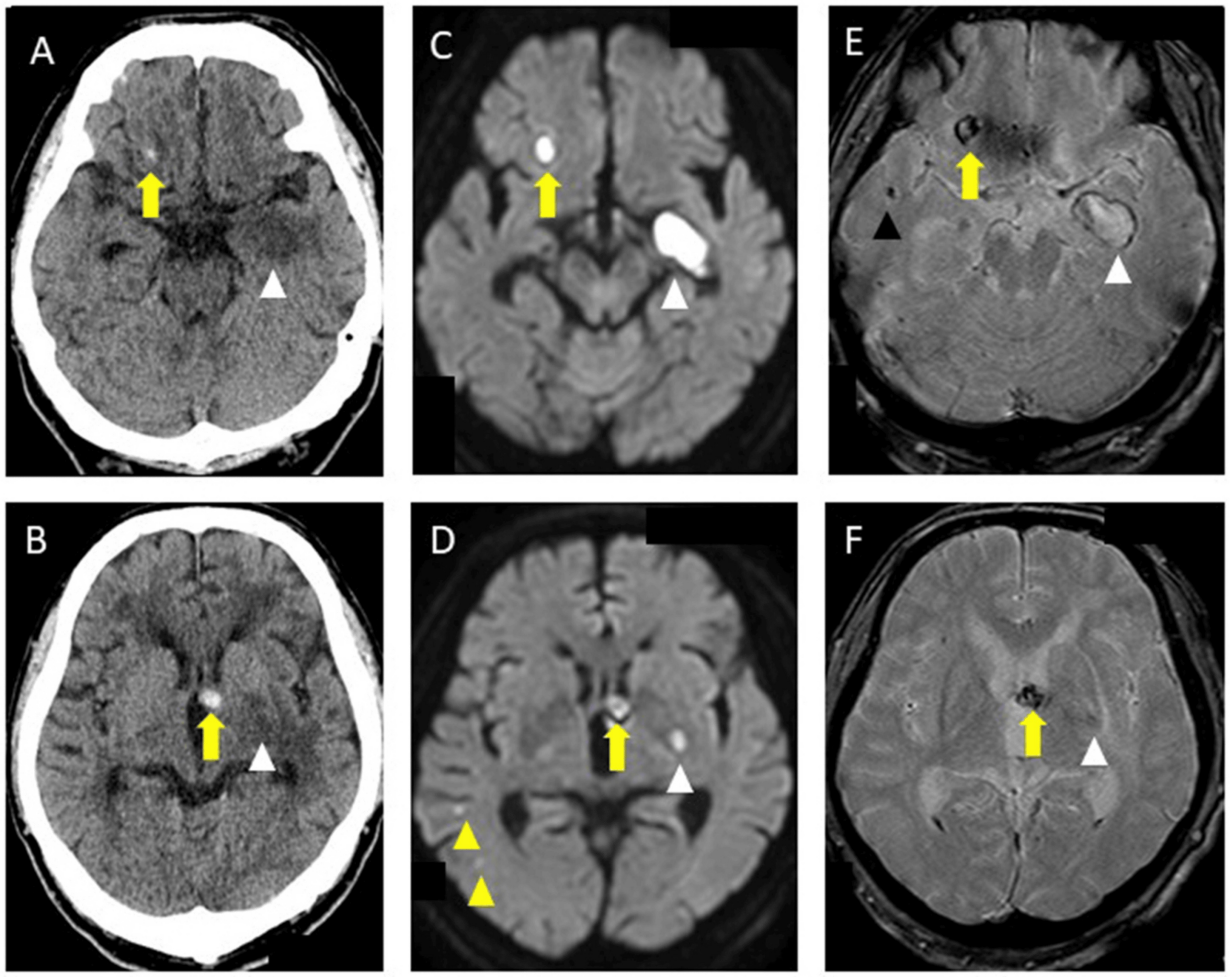
Brain computed tomography (CT), magnetic resonance imaging (MRI) diffusion-weighted imaging (DWI), and T2* imaging on day 122 A, B: Brain CT revealed acute cerebral hemorrhage in the right frontal subcortex and left internal capsule genu (yellow arrow), and new low-density area in the left temporal lobe and basal ganglia (white triangle). C, D: Brain MRI-DWI demonstrated new small high signals in the right left temporal sub-cortex of new small infarctions (yellow arrow), new high signals in the right frontal subcortex and left internal capsule genu of acute cerebral hemorrhage (yellow arrow), and new high signals in the left temporal lobe and left basal ganglia of sub acute intracerebral hemorrhage (white triangle). E, F: T2* imaging revealed new right temporal microbleeds (black triangle), annular low intensity in the right frontal subcortex and left internal capsule genu of acute cerebral hemorrhage (yellow arrow), and annular low intensity in the left temporal lobe and left basal ganglia of subacute cerebral hemorrhage (white triangle).

On day 122, the patient suddenly experienced low-grade fever (37.3℃), accompanied by a decreased consciousness level, and visited our emergency room. Neurological examination revealed a consciousness level of JCS-II-10, and slurred speech. Brain CT revealed new acute cerebral hemorrhage in the right frontal subcortex and left internal capsule genu (Figure 6A, 6B). Brain MRI-DWI, and T2* imaging showed new microinfarctions in the right temporal subcortex, new right temporal MBs, and new subacute cerebral hemorrhage in the left temporal lobe and basal ganglia (Figure 6C-6F). We immediately discontinued apixaban, initiated strong antihypertensive treatment with nicardipine for intracerebral hemorrhage, and increased lacosamide to 200 mg/day, suspecting that the patient could have complicated systemic epilepsy. Based on the criteria for diagnosing CAA-Ri [5], we also suspected possible CAA-related inflammation (CAA-Ri) as the serum ESR and CRP of the patient were positive, CSF protein levels were slightly elevated, and brain contrast MRI showed meningeal enhancement. We initiated steroid pulse therapy with intravenous methylprednisolone at 1000 mg/day for 3 days. Additionally, the steroid pulse therapy was repeated three times at 2-week intervals.　

**Figure 7:**
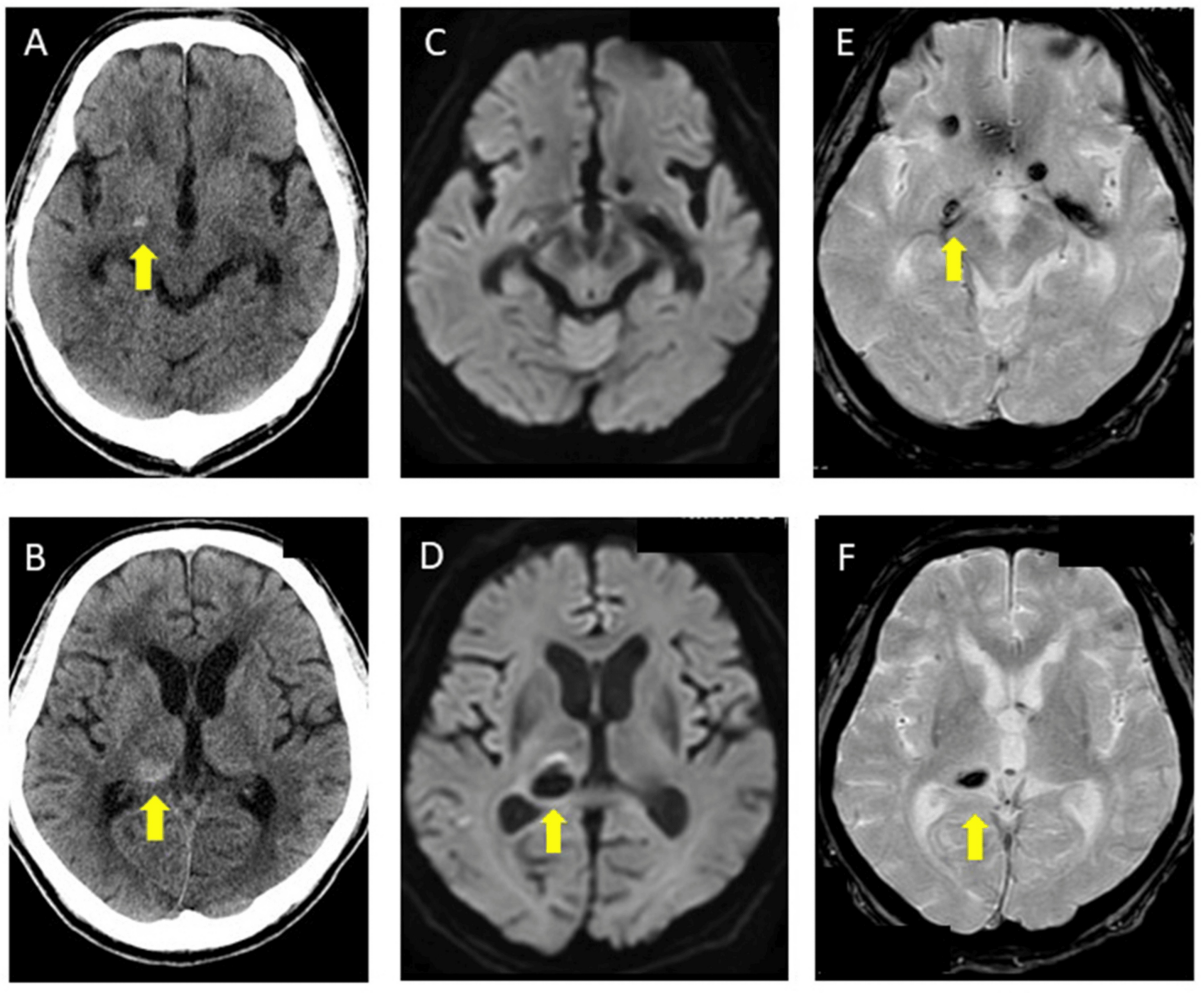
Brain computed tomography (CT), magnetic resonance imaging diffusion-weighted imaging (DWI), and T2* imaging on day 172 A, B: Brain CT showed an asymptomatic thalamic hemorrhage (yellow arrow). C, D: Brain MRI-DWI showed an asymptomatic thalamic hemorrhage (yellow arrow). E, F: T2* imaging showed an asymptomatic thalamic hemorrhage (yellow arrow).

However, the patient developed steroid-induced delirium, which we were unable to manage. Moreover, a brain CT on day 117 showed no expansion of the cerebral hemorrhage. Thus, we adjusted the medication regimen by discontinuing amlodipine at 10 mg/day and olmesartan at 40 mg/day, replacing them with sacubitril valsartan at 200 mg/day, and reinstated apixaban at 5 mg/day for AF. On day 172, brain MRI-DWI, T2* imaging, and CT revealed an asymptomatic right thalamic hemorrhage (Figure 7A-7F), leading to the discontinuation of apixaban. The patient demonstrated no further symptoms or increases in DWI high spots or MBs over the next 7 months.

**Figure 8:**
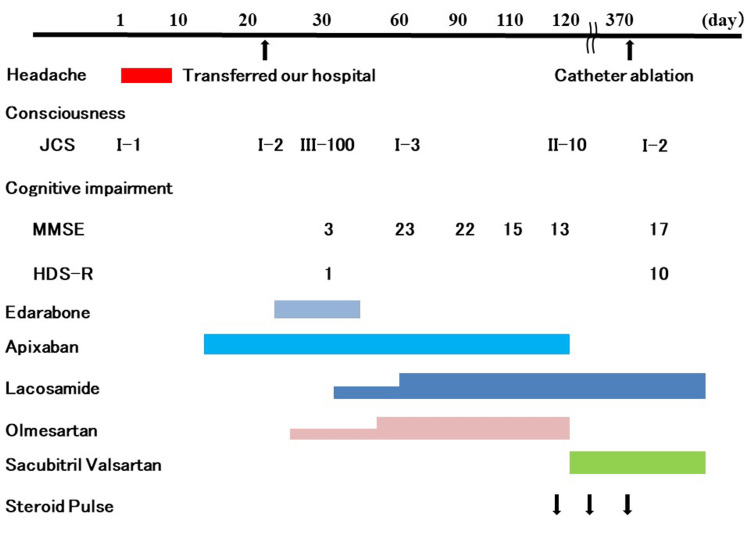
Clinical course and treatment JCS: Japan Coma Scale, MMSE: Mini Mental State Examination, HDS-R: Hasegawa's Dementia Scale-Revised (HDS-R) score.

On day 393, he underwent catheter ablation (CA) for superior pulmonary vein isolation, posterior wall isolation, superior vein, and tricuspid valve-inferior cava at B hospital. The ECG sinus rhythm returned to normal after CA. Apixaban at 10 mg/day was administered for 1 week after CA, followed by apixaban at 5 mg/day for 1 month. Apixaban was discontinued after confirming that sinus rhythm was maintained. On day 410, the Mini Mental State Examination (MMSE) score was 17/30, and the Hasegawa's Dementia Scale-Revised (HDS-R) score was 10/30. The follow-up MRI on days 425, 460, and 495 showed no increase in DWI high spots, MBs, or cSS. The patient’s progress is shown in Figure 8.

## Discussion

A definitive diagnosis of CAA previously warranted a pathological diagnosis via brain biopsy. Fortunately, it is now possible to diagnose probable and possible CAA noninvasively using MRI, as per the latest Boston Criteria 2nd Edition [1]. Accordingly, our patient received a diagnosis of CAA without a brain biopsy.

As AF and CAA are two common diseases in elderly populations, patients with CAA often also have AF, which makes it difficult to distinguish microinfarctions caused by CAA from cardiac microembolism due to AF. CAA can cause large cerebral hemorrhages, and management of CAA patients with AF can be a major therapeutic challenge when administering antithrombotic therapy to prevent secondary cerebral infarctions [2-4]. In the current case, the patient presented with various symptoms, including a decreased level of consciousness, cognitive impairment, and increasing infarctions on DWI. Initially, we suspected microembolisms due to AF and continued treatment with DOAC. However, the patient’s condition worsened progressively; infarctions, T2* imaging MBs, and cSS increased within a short period. We diagnosed that the microinfarctions in the patient were due to CAA and not due to AF, and discontinued DOAC; as a result, there was no increase in cerebral infarction or MBs on MRI, and the patient's impaired consciousness and cognitive function gradually improved. Thus, DOAC can be harmful for CAA patients with AF linked to cortical hemorrhage; thus, it is important to discontinue DOAC for microinfarctions in cases of CAA with AF.

Recently, microinfarctions with CAA have been more widely studied [6,7]. The mechanism of micro-infarctions with CAA is thought to involve the deposition of Aβ in the cerebral superficial arterioles, leading to vascular wall thickening, lumen narrowing, and endothelial and vascular smooth muscle dysfunction, impairing the local control of cerebral blood flow and causing arteriolar occlusion [6,7]. There is no evidence-based treatment for microinfarctions with CAA, although a few reports suggest that cilostazol and memantine may be effective [8-11]. It has been reported that CAA-Ri causes increasing microinfarctions more quickly than CAA [12], and CAA-Ri may have caused multiple microinfarctions in a short period in our case.

CAA-Ri is a clinically important diagnosis because many patients with CAA-Ri respond to immunosuppressive therapy [5,13]. According to the diagnostic criteria for CAA-Ri, the patient met the following criteria: Age over 40 years (68 years); Presence of one of the following clinical features: headache, decreased consciousness, focal neurological signs, or seizures, with the presentation not directly attributable to an acute intracerebral hemorrhage: symptoms included headache, decreased consciousness, hemiparesis, and seizure; MRI scans indicating white matter hyperintensities; Presence of one or more of the following cortical hemorrhagic lesions: multiple MBs and cSS; and absence of neoplastic, infectious, or other causes [5]. Additionally, our case showed slightly elevated ESR, elevated protein in the CSF, and contrast enhancement in the meninges, suggesting that they had CAA-Ri rather than CAA [13,14].

Thromboembolism is one of the most important complications of CA, and it is recommended that patients taking DOAC undergo CA without discontinuing DOAC, and DOAC be continued for 3 months after CA [15]. In this case, after a thorough discussion with the cardiologist, the patient was administered the normal dose of DOAC for 1 week after CA and then half the dose for 1 month. As a result, no new cerebral hemorrhage or cerebral infarction occurred. Furthermore, it has been reported that CA can reduce the risk of dementia in patients with AF by restoring sinus rhythm [16]. CA may be effective in managing CAA with AF. Regarding the use of DOAC for more than 3 months after CA surgery, it is recommended that DOAC be continued in patients with a CHADS 2 score of 2 or more, taking into consideration the risk of cerebral infarction recurrence due to AF recurrence [15]. DOAC was not used in the present case because the risk of cerebral hemorrhage recurrence was considered high, although the CHADS 2 score was 3. Left atrial appendage closure (LAAC) is considered in cases of CAA with AF, where anticoagulation is not possible [2-4,17]. LAAC had been shown to be non-inferior to DOAC in the long-term prevention of cerebral infarction [18]. Thus, we will consider performing LAAC if AF recurs in the future in this case.

Our study was not without limitations. First, we did not perform a brain biopsy. This decision was based upon the fact that our patient exhibited only small high DWI signal intensity lesions and no subcortical hemorrhage. Consequently, a biopsy was deemed unnecessary. Second, our search for CAA-specific biomarkers was not comprehensive. For instance, we did not measure Aβ40 and Aβ42 levels in the CSF of the patient, which have been shown to differ significantly in patients with Alzheimer's disease with CAA compared to controls [19]. Third, we lack the facility to utilize amyloid positron emission tomography (PET) imaging. Previous studies have reported that amyloid positron emission tomography (PET), especially 11C-PIB PET, is useful for CAA diagnosis [20]. Unfortunately, our institution only had access to 18F-florbetapir. However, the imaging sensitivity of 18F-florbetapir PET is poorer than that of 11C-PIB in CAA patients.

## Conclusions

We report a case of CAA with AF, challenging the treatment of microinfarctions with CAA. It is clinically important to promptly discontinue DOAC and consider CA and LAAC in cases of CAA with AF microinfarctions whenever possible.
